# A bibliometric analysis of autophagy in atherosclerosis from 2012 to 2021

**DOI:** 10.3389/fphar.2022.977870

**Published:** 2022-09-15

**Authors:** Fengwei Zhang, Ruirui Wang, Baocheng Liu, Lei Zhang

**Affiliations:** Shanghai Innovation Center of TCM Health Service, Shanghai University of Traditional Chinese Medicine, Shanghai, China

**Keywords:** Atherosclerosis, Autophagy, bibliometric, CiteSpace, VOSviewer

## Abstract

**Background:** Regulation of autophagy affects the progression of atherosclerosis. In recent years, research on autophagy in atherosclerosis has been widely concerned. However, there is no bibliometric analysis in this field.

**Objective:** The purpose of this study was to explore the general situation, hot spots, and trends of the research in this field through bibliometric analysis.

**Methods:** Articles related to autophagy in atherosclerosis from 2012 to 2021 were retrieved from the Web of Science Core Collection. VOSviewer and CiteSpace were used for data analysis and visualization of countries, institutions, authors, keywords, journals, and citations.

**Results:** A total of 988 articles were obtained in the last 10 years. The number of publications and citations increased rapidly from 2012 to 2021, especially after 2019. The most productive countries, institutions, journals, and authors were the People’s Republic of China, Shandong University, *Arteriosclerosis Thrombosis and Vascular Biology*, and Wim Martinet, respectively. The primary keywords were “oxidative stress,” “apoptosis,” “activated protein kinase,” and “inflammation.” The burst detection analysis of keywords found that “SIRT1” and “long non-coding RNA” might be regarded as the focus of future research.

**Conclusion:** This is the first bibliometric analysis of autophagy in atherosclerosis, which reports the hot spots and emerging trends. The interaction between oxidative stress and autophagy, programmed cell death, and activated protein kinases are considered to be the current research priorities. Molecular mechanisms and therapeutic target for the intervention of atherosclerosis by regulating autophagy will become an emerging research direction.

## Introduction

Atherosclerosis is a chronic disease caused by inflammation and lipid deposition (Xu et al., 2018b; Libby et al., 2019), which mainly occurs in medium and large arteries (Falk, 2006). Lipid-rich necrotic cores and vascular wall sclerosis caused by atherosclerosis will lead to thickening and stiffening of the arterial walls and narrowing of the vascular lumen (Glass and Witztum, 2001; Bartels et al., 2015). It is worth noting that atherosclerosis is the major reason of vascular diseases in the world (Herrington et al., 2016), and can lead to ischemic heart disease (Do et al., 2014), stroke (Flores et al., 2020), and peripheral vascular diseases (Alpert, 2011). According to the report on clinical practice guidelines of the American College of Cardiology, atherosclerotic cardiovascular disease is the leading cause of death worldwide and places a heavy burden on society (Arnett et al., 2019). Additionally, the incidence of atherosclerosis shows a trend in younger in the past few years (Sanz et al., 2009; Libby, 2021).

The pathogenesis of atherosclerosis is complex and diverse, and its molecular mechanism has not yet been fully elucidated (Keating et al., 2016; Wang et al., 2021). Early studies have proved that lipid metabolism disorders are the pathological basis of atherosclerosis (Keys et al., 1958). In the 80s, researchers found that a large number of lipid inclusions expand the cytoplasm into foam cells, which are the main signs of early atherosclerosis (fatty streak lesion) (Fowler, 1980; Fowler et al., 1985). Autophagy is a cellular pathway that degrades proteins and organelles by forming autophagosomes (Martinez-Lopez and Singh, 2015). Autophagy was first discovered in human liver cells in 1962 (Ashford and Porter, 1962). Recently, autophagy has been recognized as a critical participant in cellular (Heaton and Randall, 2010; Donato et al., 2018), immunity (Deretic and Levine, 2018; Ligeon et al., 2021), and body metabolism (Meijer, 2009; Kim and Lee, 2014; Li et al., 2015a). Regulation of autophagy plays a prominent role in maintaining the balance (Zhang et al., 2018) and metabolism in the body, especially in metabolic diseases such as atherosclerosis (Razani et al., 2012; Sergin et al., 2017), diabetes (Gonzalez et al., 2011; Mellor et al., 2011; Bachar-Wikstrom et al., 2013), and obesity (Liu et al., 2015; Klionsky et al., 2021). Meanwhile, previous studies have found that autophagy can regulate lipid metabolism and degrade lipid droplets through a process called lipophagy (Singh et al., 2009; Khawar et al., 2019). Dysregulation of autophagy often leads to excessive accumulation of lipids in tissues (Liu and Czaja, 2013). Indeed, autophagy is also involved in the progression of atherosclerosis in such aspects as inflammation and apoptosis (Jia et al., 2007; Shao et al., 2016). Autophagy dysfunction is often accompanied by the progress of atherosclerosis (Zhang et al., 2021a). That is to say, autophagy defect aggravates the occurrence of atherosclerosis (Razani et al., 2012; Bravo-San Pedro et al., 2017). Overall, autophagy is considered to be a potential herapeutic target that may affect the treatment of arteriosclerosis (Shao et al., 2016; Grootaert et al., 2018b).

As mentioned above, considerable research efforts have been directed at autophagy in atherosclerosis. The precise regulation of autophagic flux provides new ideas for developing anti-atherosclerosis drugs (Hua et al., 2022). However, a large number of experimental and clinical studies are required to determine their potential utility in practice. The application of autophagy in human diseases is still limited (Henderson et al., 2021; Mizushima et al., 2021). Besides, the monitoring methods for autophagy are constantly refined (Klionsky et al., 2012). Therefore, the research on topics evolution and frontier trends has far-reaching significance. As far as we know, there is no bibliometric analysis of this research topic.

Bibliometrics is an emerging knowledge synthesis approach that identifies publications’ quantitative and qualitative attributes (Zhang et al., 2020) and explores prominent research trends in research areas (Telis et al., 2016). With the explosive growth of scientific research, the metrological analysis of publications has become increasingly important (Kim and Chen, 2015). In recent years, computational and visual analytic technologies have provided advanced methods for bibliometric analysis in specific fields (Chen and Song, 2019). CiteSpace is an application based on the JAVA platform designed by Professor Chaomei Chen (Chen, 2006). It can identify research hot spots and leading edges through the algorithm and then display them by the view mode. VOSviewer is a tool developed by Van Eck and Waltman of Leiden University in the Netherlands, which can be used to build knowledge maps and visual bibliometric networks (Waltman et al., 2010). Therefore, through bibliometric analysis based on published studies related to autophagy in atherosclerosis, we intend to answer the following research questions: How about the scientific productivity and distribution in this field? What are the current themes being explored in this field? Our study aims to reveal the research distribution, research hot spots, and development trend of autophagy in atherosclerosis. Based on our research, researchers and policymakers can better understand the research frontiers in molecular mechanisms, gene regulation, and benefit-to-risk balance. Moreover, this allows doctors to stay up to date on the most current evidence in key clinical areas and then make more informed decisions. In addition, this also provides new ideas for targeted drug discovery and development.

## Materials and methods

### Data collection

Data from this study were extracted from the Web of Science Core Collection (WoSCC). The search formula was set as “{[TS = (atherosclerosis)] AND [TS = (autophagy OR autophage OR autophagocytosis)] AND PY = (2012–2021)}” Conditions were set for all articles published between 1 January 2012 and 31 December 2021, with no restrictions on language and article type. A total of 988 articles were obtained, and all the records and cited articles were exported in plain text files. The exported records were named after “download_*.txt” for subsequent analysis.

### Data analysis

This study performed bibliometric and visual analyses based on Microsoft Excel 2016, CiteSpace 5.8. R3 (64 bit), and VOSviewer 1.6.18.

Microsoft Excel was used to analyze annual publications and citations, literature type, country distribution, and language.

Based on CiteSpace, we conducted the collaboration network analysis of countries and institutions, co-occurrence analysis of keywords, and co-citation analysis of references. Each node in the visual map represents the country, institution, author, and other subjects to which it belongs. Its size indicates the frequency of appearance. In networks, betweenness centrality measures how central a vertex is about shortest paths (Ma et al., 2020). The nodes with a betweenness centrality >0.1 are still represented by a purple ring and are considered crucial nodes (Chen, 2006). Meanwhile, the line reflects the connection between different subjects such as cooperation, reference, concordance, etc. In addition, we also performed keyword burst detection analysis to identify fast-growing topic keywords and emerging areas. In this study, the following parameters were selected for setting: Time slice (2012–2021), years per slice (1), and selection strategy (g-index, *k* = 25).

VOSviewer was used to analyze and visually display large-scale data samples in intuitive forms such as overlay visualization, network visualization, and density visualization. In this study, VOSviewer was used to obtain the co-occurrence network of authors, authors, journals, keywords and references, as well as the density map of co-cited journals.

## Results

### General data

**FIGURE 1:**
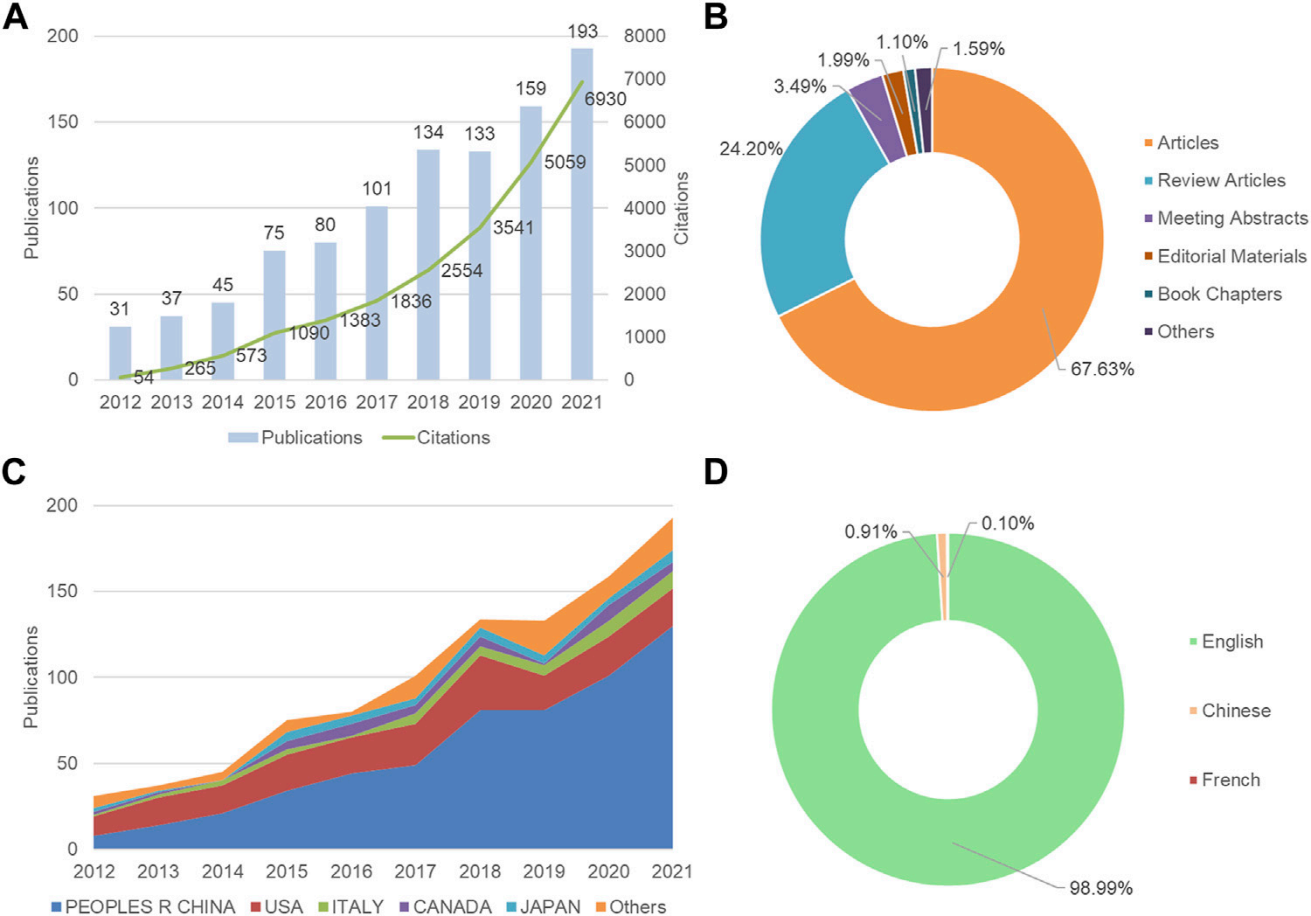
Overview of autophagy in atherosclerosis studies from 2012 to 2021. **(A)** The annual distribution of publications and citations; **(B)** Type of article; **(C)** The trend of publications of the five most productive countries/regions per year; **(D)** Language of publications.

A total of 988 articles were retrieved from 2012 to 2021. The annual number of publications has generally increased from 2012 onwards, except in 2019 (Figure 1A). A total of 17,555 articles were cited, with the cumulative total citations of 24,685 times and an average of 24.98 times per article. H-index was 75, which was considered possible to assess the scientific impact of the field. As shown in Figure 1B, articles and reviews accounted for 67.63% and 24.20% of all literature materials, respectively. Figure 1C shows the annual number of communications from the top five countries or regions. Starting in 2014, the People’s Republic of China surpassed the United States as the country with the most publications per year. In terms of language, the majority of the articles were published in English, in addition to Chinese and French (Figure 1D).

### Countries and institutions analysis

**FIGURE 2:**
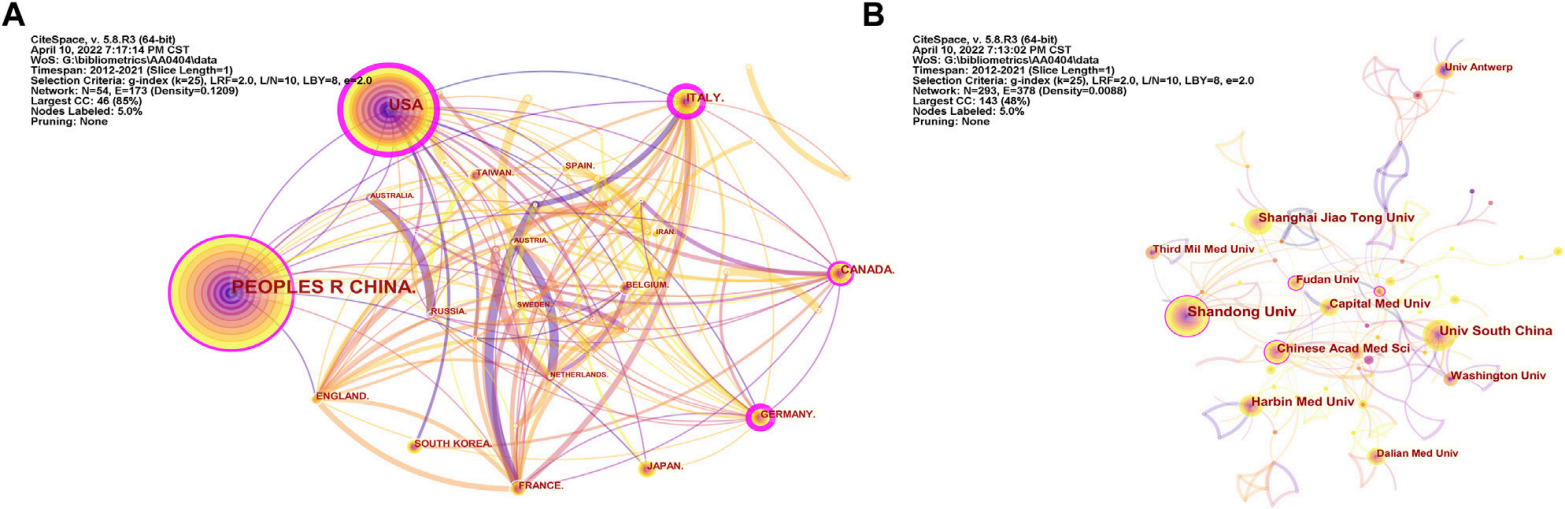
Countries and Institutions Analysis. **(A)** Analysis of national cooperation based on CiteSpace visual map; **(B)** Analysis of institutional cooperation based on CiteSpace visual map. Each node represents a country or institution, and its size reflects the number of contributions. The connections between nodes reflect the relationships between them.

There were 293 institutions from 54 countries that published literature related to autophagy in atherosclerosis. Among them (Figure 2A), the most productive countries were the People’s Republic of China (532), the United States (192), Italy (43), Canada (39), Germany (31) and Japan (31). The United States had the highest centrality (0.36) with purple circles, indicating that it had advantages in terms of national cooperation and influence (Table 1). Most countries with high centrality are European countries, while some Asian countries still lack international cooperation despite publishing more articles. As shown in Figure 2B and Table 2, Shandong University has 43 articles with the most significant number of publications. The second most important ones were Nanhua University (30), Shanghai Jiaotong University (26), Harbin Medical University (26), and the Chinese Academy of Medical Sciences (21). The top 10 productivity institutions are universities or research institutes, and nine are from China. These results show that China is at the forefront of the world.

**TABLE 1:** Top 10 countries in terms of publications and centrality.

Rank	Count	Centrality	Country	Rank	Centrality	Count	Country
1	532	0.14	China	1	0.36	192	United States
2	192	0.36	United States	2	0.29	43	Italy
3	43	0.29	Italy	3	0.24	31	Germany
4	39	0.18	Canada	4	0.18	39	Canada
5	31	0.24	Germany	5	0.14	532	China
6	31	0	Japan	6	0.09	9	Austria
7	26	0.06	France	7	0.07	11	Netherlands
8	24	0	South Korea	8	0.06	26	France
9	21	0.05	England	9	0.06	12	Iran
10	20	0.01	Belgium	10	0.06	4	Israel

**TABLE 2:** Top 10 institutions with the most publications.

Rank	Count	Centrality	Institution	Country
1	43	0.1	Shandong University	China
2	30	0.04	University of South China	China
3	26	0.05	Shanghai Jiao Tong University	China
4	26	0.03	Harbin Medical University	China
5	21	0.2	Chinese Academy of Medical Sciences	China
6	21	0.02	Capital Medical University	China
7	20	0.14	Fudan University	China
8	17	0.02	University of Washington	United States
9	15	0.02	Third Military Medical University	China
10	14	0.01	Dalian Medical University	China

### Analysis of author and co-cited author

**FIGURE 3:**
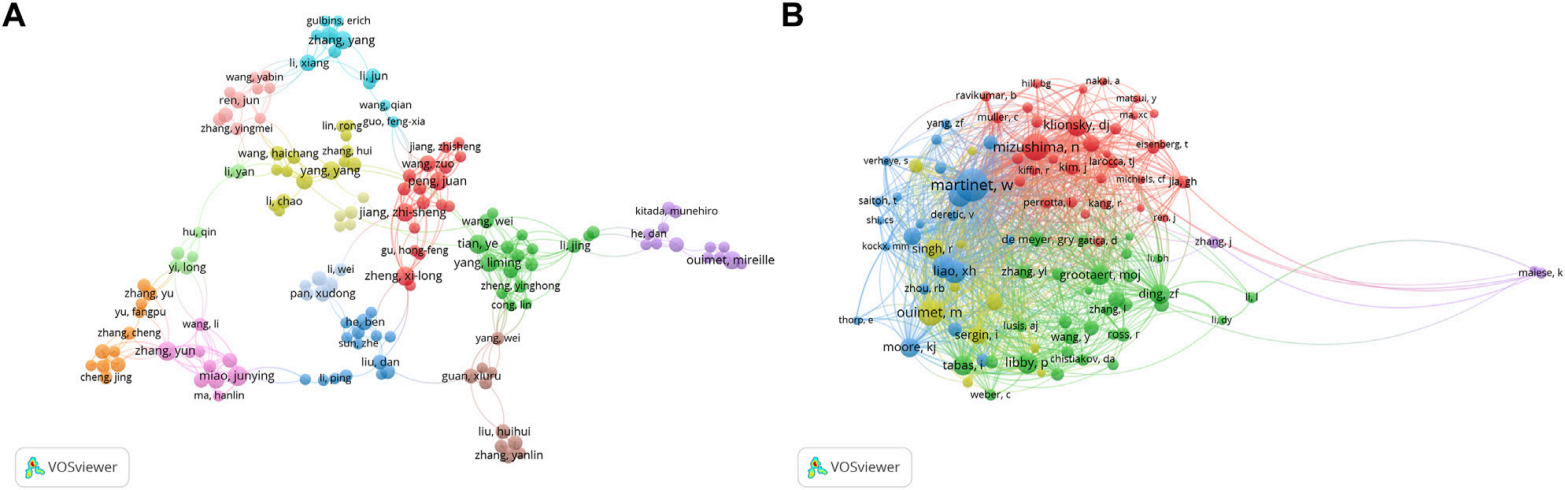
Analysis of author and cited author. **(A)** Author co-authorship network analysis based on VOSviewer visualization map; **(B)** Author co-citation network analysis based on VOSviewer visualization map. Different color clustering reflects the cooperative relationship between authors.

Three hundred ninety-one authors contributed to the study of autophagy in atherosclerosis, with Wim Martinet, Guido R Y De Meyer, and Babak Razani as the most prolific authors. The co-authorship network is depicted in Figure 3A. Wim Martinet published 14 articles on the subject at the University of Antwerp, Belgium. He was involved in drafting guidelines for the use and interpretation of assays for monitoring autophagy (third edition) (Klionsky et al., 2016), which were cited 3,532 times. Authors in different color clusters represent different closely related groups. In the red cluster, Peng J., Wang Z., and Jiang Z. S. were all from the Institute of Cardiovascular Disease, University of South China. They work together to regulate autophagy with tet methylcytosine dioxygenase 2 (TET 2) (Li et al., 2015b). And prove that the up-regulation of TET 2 can inhibit atherosclerosis (Peng et al., 2016). The green clusters were represented by Yang L. M., Tian Y., Zheng Y. H., and Cong L. from Harbin Medical University. In their research, photodynamic therapy (Han et al., 2018), sonodynamic therapy (Jiang et al., 2017) and electric stimulation (Cong et al., 2020) have been proved to promote autophagy and inhibit inflammation.

The authors with the highest co-citations and the top 10 most cited authors are presented in Figure 3B and Tables 3, respectively. Among them, 14 authors’ articles were cited more than 100 times, indicating that their research enjoyed a high reputation and influence. Red clusters such as Klionsky DJ and Mizushima N et al. were mainly involved in the compilation of guidelines for autophagy detection (Klionsky et al., 2016). They provided an overview of the role of autophagy dysfunction in the pathogenesis of major human diseases (Klionsky et al., 2021). In the blue cluster, Martinet W. and Verhoey S. found an association between plaque temperature and vulnerability in atherosclerotic plaques (Van De Parre et al., 2007; Van De Parre et al., 2008). They also explored the significance of cryotherapy in enhancing plaque stability in animal experiments (Verheye et al., 2016). The authors from the green cluster focused on the role of non-lipid factors in atherosclerosis, such as inflammation (Hansson et al., 2015), immunity (Packard et al., 2008), and enzymatic reactions (Grootaert and Bennett, 2022). The above scholars’ research in this field has been recognized, and they have made contributions to the exploration of the mechanism of atherosclerosis and the development of new treatments.

**TABLE 3:** Top 10 most productive authors distributed by publications and citations.

Rank	Count	Author	Rank	Count	Cited author
1	14	Wim Martinet	1	239	Martinet W.
2	12	Guido R. Y. De Meyer	2	207	Mizushima N.
3	10	Babak Razani	3	195	Liao X. H.
4	8	Zufeng Ding	4	192	Ouimet M.
5	8	Shijie Liu	5	185	Razani B.
6	8	Jawahar L. Mehta	6	153	Levine B.
7	8	Xianwei Wang	7	140	Libby P.
8	6	Ismail Sergin	8	132	Klionsky D. J.
9	6	Zhisheng Jiang	9	118	Grootaert Moj
10	6	Juan Peng	10	118	Moore K. J.

### Analysis of core journals

**FIGURE 4:**
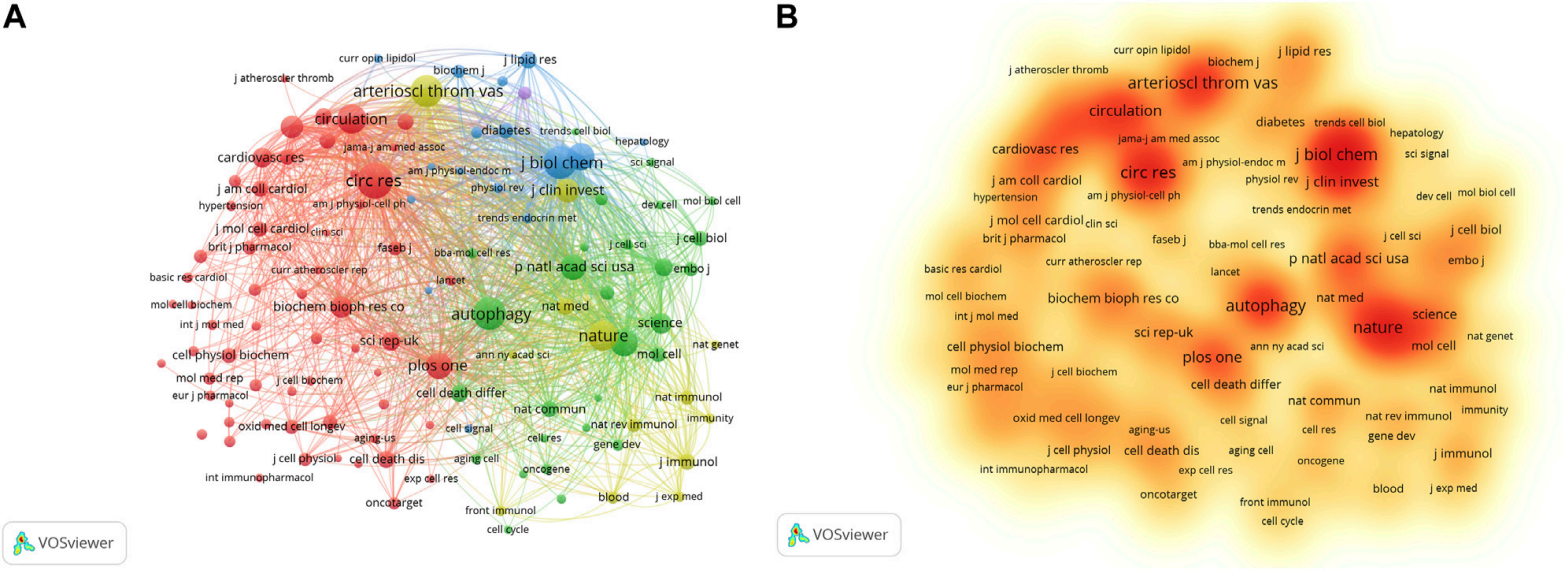
Analysis of co-cited journals based on VOSviewer visual map. **(A)** Network visualization; **(B)** Density visualization. Each node on the map is filled with a color based on the element density. Journals with high citation frequencies are indicated in red.

The researchers published literature in 378 journals. The top 10 journals are presented in Table 4. *Arteriosclerosis Thrombosis and Vascular Biology* has the largest number of publications, with 24 articles. Followed by *Frontiers in Pharmacology* (22), *Oxidative Medicine and Cellular Longevity* (22), and *Autophagy* (21). About one-fifth of the literature was from the top 10 journals. The average impact factor (IF) of the top 10 journals was 6.60, and the average H-index was 142.8. Cell biology, biochemistry, and molecular biology are the main journal categories. A total of 118 journals were cited more than 100 times. The top three co-cited journals were *Journal of Biological Chemistry*, *Circulation Research*, and *Autophagy* (Figures 4A,B).

**TABLE 4:** Top 10 journals with most publications.

Rank	Count	Jounals	IF	H-index	Country
1	24	Arteriosclerosis Thrombosis and Vascular Biology	8.311	251	United States
2	22	Frontiers in Pharmacology	5.810	62	Switzerland
3	22	Oxidative Medicine and Cellular Longevity	6.543	66	United States
4	21	Autophagy	16.016	121	United States
5	20	Biochemical and Biophysical Research Communications	3.575	243	United States
6	20	International Journal of Molecular Sciences	5.923	114	United States
7	18	Scientific Reports	4.379	149	England
8	17	Molecular Medicine Reports	2.952	43	Greece
9	16	Cell Death & Disease	8.469	111	England
10	16	PloS One	3.24	268	United States

### Analysis of keywords and hot spots

**FIGURE 5:**
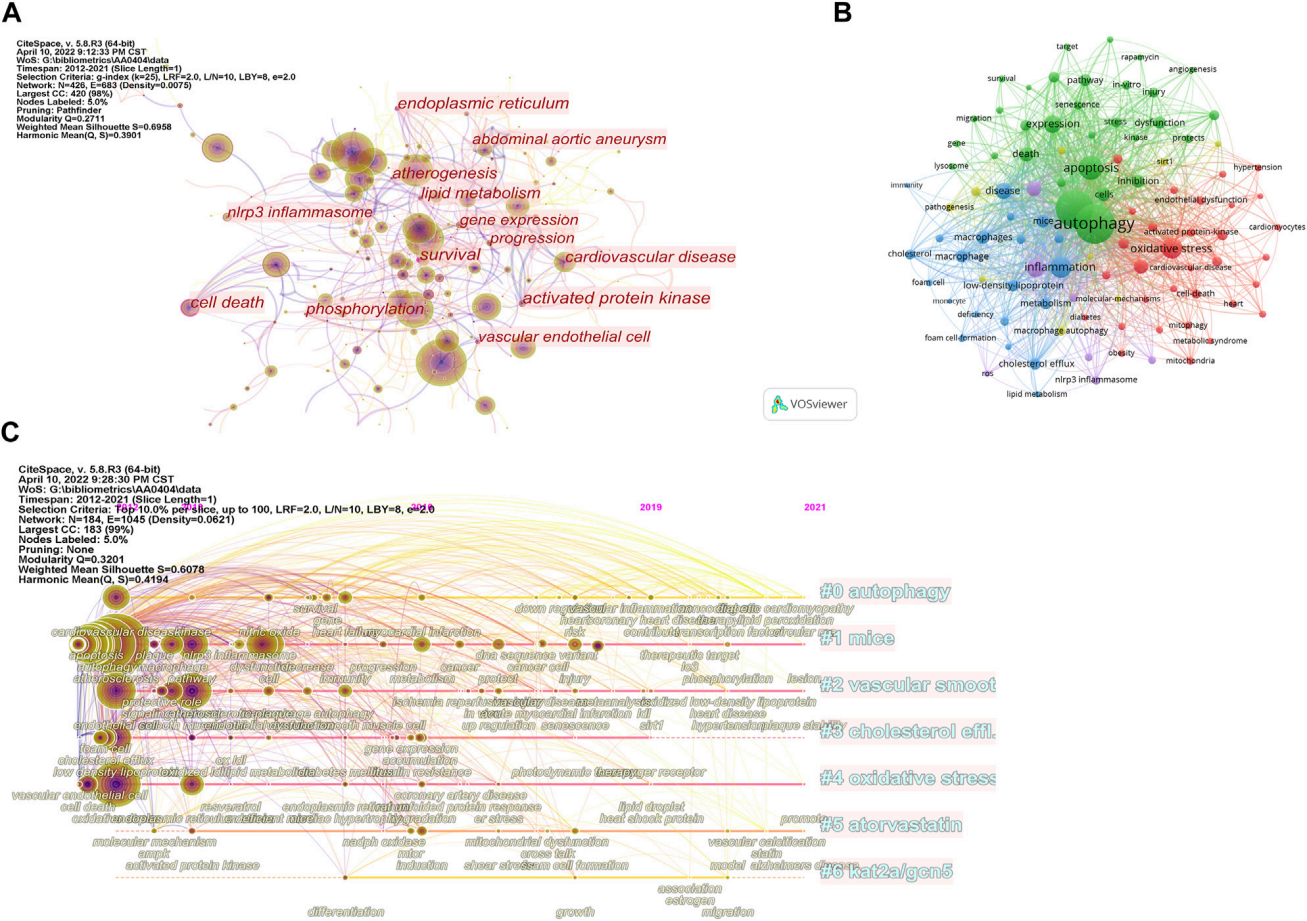
Analysis of keywords. **(A)** Analysis of keywords based on CiteSpace visual map; **(B)** Analysis of keywords based on VOSviewer visual map; **(C)** Timeline view of keywords.

As shown in Figure 5A and Table 5, there are 21 keywords that have appeared more than 50 times, among which “atherosclerosis,” “autophagy,” “oxidative stress,” “apoptosis,” and “activation” were at the top. “Cell death” was the keyword with the highest degree of centrality (0.31), appearing 31 times. Then there are “survival,” “activated protein kinase,” and “endoplasmic reticulum”. In Figure 5B, the green cluster is mainly composed of “autophagy,” “apoptosis,” and “death.” This cluster focuses on the mechanisms involved in programmed cell death. The blue clusters were mainly composed of “inflammation,” “macrophages,” “foam cells,” and “cholesterol.” The accumulation of fat in macrophages and the formation of foam cells are early signs of atherosclerosis. The red cluster is represented by “oxidative stress,” “endothelial dysfunction,” and “cardiovascular disease.” Oxidative stress refers to the imbalance between oxidation and antioxidation *in vivo*, which often leads to endothelial dysfunction and cardiovascular disease.

**TABLE 5:** Top 10 keywords in terms of publications and centrality.

Rank	Count	Centrality	Keywords	Rank	Centrality	Count	Keywords
1	299	0	Atherosclerosis	1	0.31	45	Cell death
2	235	0.03	Autophagy	2	0.21	19	Survival
3	166	0.07	Oxidative stress	3	0.19	28	Activated protein kinase
4	158	0.02	Apoptosis	4	0.18	11	Endoplasmic reticulum
5	131	0.02	Activation	5	0.16	25	Nlrp3 inflammasome
6	130	0.12	Expression	6	0.16	24	Lipid metabolism
7	115	0.01	Disease	7	0.16	20	Gene expression
8	112	0.14	Mechanism	8	0.16	15	Atherogenesis
9	104	0.02	Inflammation	9	0.15	28	Protective role
10	100	0.01	Smooth muscle cell	10	0.14	112	Mechanism

The timeline visualization of the keywords is shown in Figure 5C, and the first seven clusters of related high-frequency words are shown in Table 6. “Autophagy,” “mice,” and “vascular smooth muscle cells” were large clusters with multiple articles. Vascular smooth muscle cells and oxidative stress have been widely concerned by different researchers. Acetylation plays a vital role in the regulation of autophagy, with lysine acetyltransferase 2A (KAT 2A/GCN 5) gaining attention after 2015.

**TABLE 6:** Top seven clusters of kewords.

ClusterID	Size	Year	Label (LLR)
#0	42	2018	Autophagy
#1	42	2014	Mice
#2	31	2017	Vascular smooth muscle cells
#3	26	2014	Cholesterol efflux
#4	19	2015	Oxidative stress
#5	18	2017	Atorvastatin
#6	5	2018	Kat2a/gcn5

Keywords burst can reflect research hotspots and trends in this field. Table 7 shows the ranking of keywords with the strongest citation burst according to chronological order. The burst strength indicates the intensity of the word frequency change in a period of time (Shen et al., 2019). “Plaque,” “ox ldl,” and “oxidized ldl” were the three strongest burst keywords. These keywords may be the core keywords of autophagy in atherosclerosis, and suggest that the pathological changes of atherosclerosis are the major concerns. From 2012 to 2015, researchers focused on endothelial cells and atherosclerosis formation. Oxidized low-density lipoprotein formed by oxidative modification of natural low-density lipoprotein received attention after 2013. In recent years, senescence and the related enzyme SIRT1 have been considered new directions for studying autophagy and atherosclerosis.

**TABLE 7:** Top 18 Keywords with the strongest citation bursts.

Keywords	Year	Strength	Begin	End	2012–2021
SIRT1	2012	2.78	2019	2021	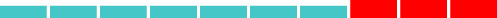
Long noncoding rna	2012	2.5	2019	2021	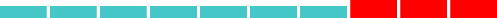
Vascular inflammation	2012	2.5	2019	2021	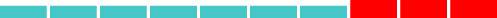
Senescence	2012	2.51	2018	2019	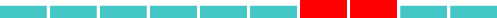
*In vivo*	2012	2.66	2017	2018	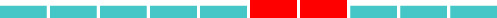
Ischemia reperfusion injury	2012	2.58	2017	2018	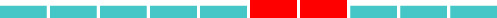
Metabolic syndrome	2012	3.13	2016	2018	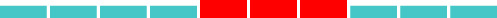
Homeostasis	2012	2.43	2016	2018	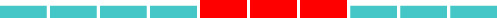
Identification	2012	3.26	2015	2016	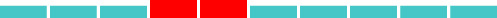
ox ldl	2012	3.36	2014	2015	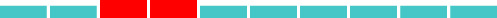
Survival	2012	2.49	2014	2015	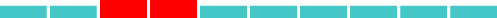
Plaque	2012	3.6	2013	2017	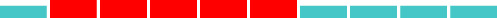
Oxidized ldl	2012	3.31	2013	2017	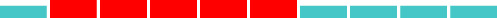
Unfolded protein response	2012	2.68	2013	2017	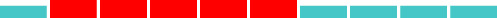
Nadph oxidase	2012	2.6	2013	2016	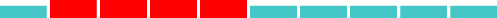
Artery endothelial cell	2012	2.42	2013	2014	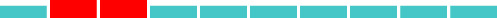
Vascular endothelial cell	2012	2.71	2012	2013	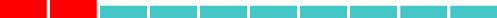
Atherogenesis	2012	2.64	2012	2015	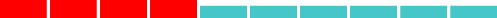

### Analysis of cited articles and co-cited references

**FIGURE 6:**
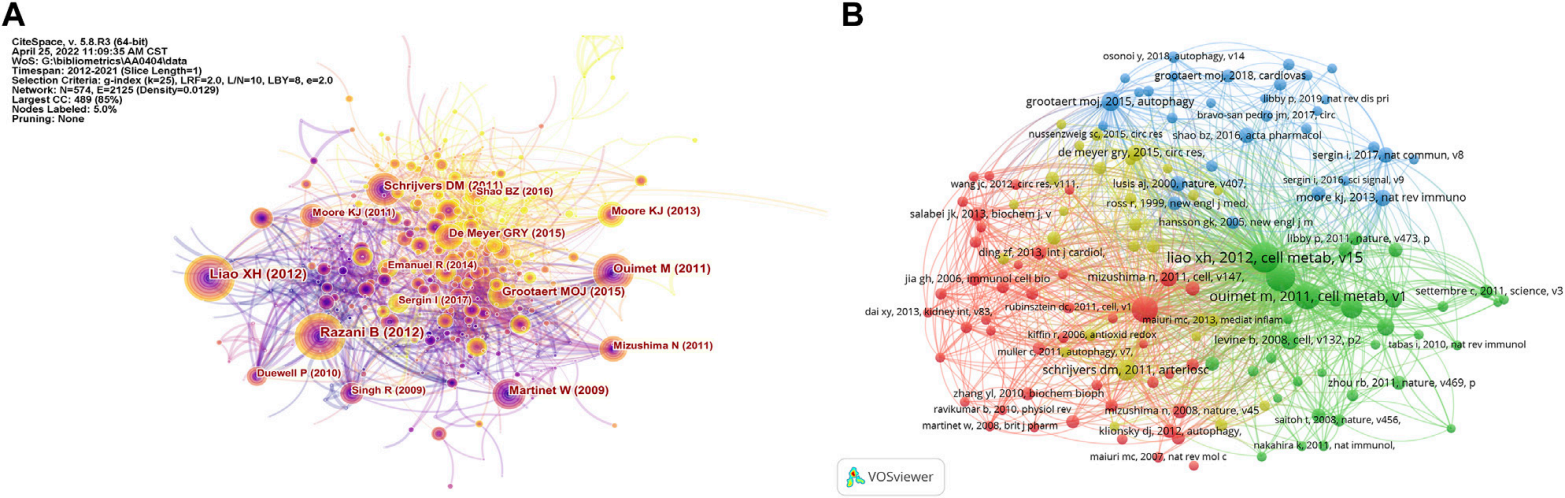
Analysis of cited articles and co-cited references. **(A)** Analysis of cited articles based on CiteSpace visual map; **(B)** Analysis of co-cited references based on VOSviewer visual map.

A total of 142 articles were cited more than ten times. Figures 6A,B show the distribution of cited articles and co-cited references for the study of autophagy in atherosclerosis. Among them, the articles with the most frequent citation were contributed by Liao X. H., Razani B., Ouimet M., Martinet W., and Grootaert M. O. J. (Table 8). “Macrophage Autophagy Plays a Protective Role in Advanced Atherosclerosis” was the most commonly cited article, published by Liao X. H. in 2012 (Liao et al., 2012). This study found that blocking autophagy could intensify cell death and promote atherosclerotic plaque necrosis. Meanwhile, the regulation of autophagy may be a potential target to inhibit the development of atherosclerosis. These studies have established the correlation between autophagy and atherosclerosis by anti-inflammation (Razani et al., 2012), promoting cholesterol efflux (Ouimet et al., 2011), and stabilizing plaque (Schrijvers et al., 2011). Besides, the work of these researchers was frequently co-cited. In addition, the articles in the top three most highly cited articles were all from the same journal (*Cell Metabolism*). This indicates that in the field of metabolic biology, autophagy and atherosclerosis are highly correlated.

**TABLE 8:** Top 10 most cited literatures.

Rank	Count	Centrality	Title	Author	Journal	Year	Volume	Page	DOI
1	162	0.04	Macrophage autophagy plays a protective role in advanced atherosclerosis	Liao X. H.	Cell Metab	2012	15	545	10.1016/j.cmet. 2012.01.022
2	154	0.05	Autophagy links inflammasomes to atherosclerotic progression	Razani B.	Cell Metab	2012	15	534	10.1016/j.cmet. 2012.02.011
3	114	0.08	Autophagy regulates cholesterol efflux from macrophage foam cells via lysosomal acid lipase	Ouimet M.	Cell Metab	2011	13	655	10.1016/j.cmet. 2011.03.023
4	84	0.02	Autophagy in atherosclerosis: a cell survival and death phenomenon with therapeutic potential	Martinet W.	Circ Res	2009	104	304	10.1161/CIRCRESAHA.108.188318
5	76	0.06	Defective autophagy in vascular smooth muscle cells accelerates senescence and promotes neointima formation and atherogenesis	Grootaert M. O. J.	Autophagy	2015	11	2014	10.1080/15548627.2015.1096485
6	73	0.03	Autophagy in atherosclerosis: a potential drug target for plaque stabilization	Schrijvers D. M.	Arterioscl Throm Vas	2011	31	2787	10.1161/ATVBAHA.111.224899
7	59	0.02	Macrophages in atherosclerosis: a dynamic balance	Moore K. J.	Nat Rev Immunol	2013	13	709	10.1038/nri3520
8	59	0.07	Autophagy in vascular disease	De Meyer GRY	Circ Res	2015	116	468	10.1161/CIRCRESAHA.116.303804
9	53	0.02	Exploiting macrophage autophagy-lysosomal biogenesis as a therapy for atherosclerosis	Sergin I.	Nat Commun	2017	8	15750	10.1038/ncomms15750
10	53	0.01	The roles of macrophage autophagy in atherosclerosis	Shao B. Z.	Acta Pharmacol Sin	2016	37	150	10.1038/aps. 2015.87

## Discussion

### General information

In this study, we analyzed the literature related to autophagy in atherosclerosis in the past decade by bibliometric methods. A total of 988 publications from the WoSCC were obtained. According to our results, annual publications and citations increased significantly from 2012 to 2021, especially after 2019. The People’s Republic of China, with the largest number of publications, has greatly contributed to the research on autophagy in atherosclerosis. In addition, nine of the top 10 prolific institutions were from the People’s Republic of China, such as Shandong University, the University of South China, and Shanghai Jiaotong University. This indicates that autophagy has gained increasing attention from Chinese research institutions in the study of atherosclerosis mechanisms in recent years. However, countries from North America and Europe had a higher centrality ranking than countries from other regions, indicating that they attached more importance to international exchanges and cooperation. Although China is developing rapidly and occupying a dominant position in this field, its regional collaboration and academic influence still need to be improved. The above shows that the current research is imbalanced in international distribution.

Wim Martinet is the author with the most published articles and the most citations in this field. He has long been engaged in research on cardiovascular and peripheral vascular diseases, pharmacology, and cell biology. In the aspect of autophagy detection, he proposed the application of LC3 immunostaining *in situ* measurement of autophagy (Martinet et al., 2006) and established the appropriate methods to monitor it (Klionsky et al., 2016). He believed that targeted treatment of atherosclerosis by regulating the level of autophagy was of great significance (Grootaert et al., 2018a). In addition, he evaluated the advantages and disadvantages of macrophage autophagy as a drug target for plaque stabilization (Martinet et al., 2013; Martinet et al., 2019). Rajat Singh is also a highly influential author whose literature is widely cited. His study published in *Science* in 2009 illustrated the role of autophagy in lipid metabolism (Singh et al., 2009). Yoshinori Ohsumi was awarded the 2016 Nobel Prize in Physiology or Medicine for his outstanding achievements in the autophagy function of cells (Maruzs et al., 2019). His research has deepened our understanding of the function of autophagy and its effects on disease and health (Ohsumi, 2014). The above-mentioned authors enjoy a high academic reputation, because their research has accumulated valuable experience in clarifying the mechanisms of autophagy and the prevention and treatment of atherosclerosis.

A journal’s IF is calculated by comparing the average number of citations each paper received during the previous 2 years (Garfield, 2006; Loscalzo, 2011). We usually think that the IF of a journal is closely related to the influence of articles from it (Callaham et al., 2002). Among the ten journals with the largest number of published articles, *Autophagy* had the highest IF (16.016). *Autophagy* was founded in 2005, and it has become an authoritative journal in this field. *Arteriosclerosis Thrombosis and Vascular Biology* was the most prolific journal, and its IF was 8.311. This journal was sponsored by the American Heart Association and focused on vascular diseases related to arteriosclerosis and thrombosis. In addition, most active journals belong to specialized categories. Some multi-disciplinary journals, such as *Scientific Reports* and *PloS One*, also reported high-quality research on autophagy in atherosclerosis. Autophagy in atherosclerosis is a young and growing research topic involving multiple categories such as molecular biology, cell biology, biochemistry and medicine. The development of this research topic was published in journals from different disciplines, indicating that the pathogenesis and treatment of atherosclerosis received extensive attention from researchers. Besides, multi-disciplinary journals have a broader readership (Bolli, 2019), which is more conducive to cross-disciplinary collaboration.

### Hot spots and frontiers

Based on keyword analysis, we identified some of the most important hot spots from autophagy in atherosclerosis in the past decade, including: 1) Interaction between oxidative stress and autophagy (Perrotta and Aquila, 2015), including endothelial cell autophagy defect caused by excessive oxidative stress reaction (Carresi et al., 2021), formation and degradation of autophagosomes of organelles such as endoplasmic reticulum and mitochondria during oxidative stress (Mei et al., 2015), endothelial dysfunction and vascular aging mechanism (Tesauro et al., 2017), and the participation of endogenous antioxidant enzymes in the regulation of lipocyte phagocytic flux (Jeong et al., 2018). 2) Different forms of programmed cell death, such as pyroptosis, necroptosis, and apoptosis. The autophagy defect will enhance cell death and the progression of atherosclerosis (Fang et al., 2021; Shan et al., 2021). Stimulation of vascular smooth muscle through autophagy can maintain cell survival and function (Grootaert et al., 2018a), and stabilize vulnerable and ruptured plaques (Lin et al., 2021). 3) Activated protein kinases, especially in AMPK (Ou et al., 2018), PI3K/Akt (Pi et al., 2021), and MAPK(Mei et al., 2012; Yang et al., 2020) signaling pathways. 4) Endoplasmic reticulum stress and inflammatory activation (Ji et al., 2019), and in particular the activation of NLRP3 inflammasome (Hoseini et al., 2018; Chen et al., 2019).

After the burst detection analysis of keywords, we believe that sirtuins and long non-coding RNA (lncRNA) are emerging trends and development directions in the future. Sirtuins are closely related to aging, lifespan, and metabolism (Haigis and Sinclair, 2010; Lai, 2013), and have beneficial effects on cardiovascular disease (Kane and Sinclair, 2018). The most widely studied enzyme is sirtuin 1(SIRT1) (Xu et al., 2018a), which is a deacetylase that depends on nicotinamide adenine dinucleotide. SIRT1 is considered a promising new target for treating cardiovascular disease (Prola et al., 2017). It was found that activation of SIRT1 can inhibit autophagy-dependent ferroptosis (Su et al., 2021), and alleviate coronary atherosclerosis in mice (Li et al., 2022). On the contrary, inhibition of SIRT1 leads to impaired autophagy, which in turn aggravates atherosclerosis (Yang et al., 2017). Some natural products such as Araloside C. (Luo et al., 2020), Salidroside (Zhu et al., 2019), and Berberine (Zheng et al., 2021) have potential anti-atherosclerosis effects by targeting SIRT1 and increasing its expression to regulate autophagy. However, the SIRT1-autophagy axis in ameliorating atherosclerosis needs to be further explored in subsequent studies. It is worth noting that autophagy plays a dual role in the progression of atherosclerosis. Specifically, autophagy at a basal level in the early stages of atherosclerosis is considered beneficial, but excessive autophagy will produce a cytotoxic effect (Klionsky et al., 2016; Ren et al., 2020). LncRNA participates in the formation of atherosclerosis by regulating autophagy in endothelial cells, smooth muscle cells, and macrophages (Ren et al., 2020). Studies have shown that lncRNA can be associated with cholesterol deposition and macrophage-mediated inflammation (Yan et al., 2020), which will provide a reference for antisense oligonucleotide therapy of atherosclerosis (Graham et al., 2017; Kim et al., 2019). Exosome is a vital carrier to maintain cell homeostasis and transfer intercellular information (Tian et al., 2019). Exosomal lncRNA in the peripheral circulation may be a potential biomarker for the diagnosis of atherosclerosis (Yuan and Huang, 2021). In addition, exosomal lncRNA has essential value in assessing the prognosis of atherosclerotic stroke (Wang et al., 2017; Zhang et al., 2021b). Furthermore, atherosclerosis is caused by vascular inflammation (Mahmoud et al., 2016). Inducing autophagy to reduce the inflammation of vascular endothelial cells is also considered as a hot research topic (Chen et al., 2013; Meng et al., 2021). These results indicate that molecular mechanisms, therapeutic strategies, and disease evaluation will become hot spots for future research.

### Limitations

There are a few potential limitations to this study. First, the data for bibliometric analysis were only from the WoSCC. Second, our search strategy may not cover all the research in this field. Third, some publication types, such as corrections and retractions, can interfere with the results of data analysis. Fourth, our analysis may miss the latest research advances due to the rapid expansion.

## Conclusion

As far as we know, this study is the first comprehensive bibliometric analysis of autophagy in atherosclerosis from 2012 to 2021. By using CiteSpace and VOSviewer, we identified the knowledge distribution characteristics of autophagy in atherosclerosis. “Oxidative stress,” “apoptosis,” “activated protein kinase,” and “inflammation” are hot topics in this field. In addition, targeted therapy for atherosclerosis and the development of new drugs based on autophagy regulation will be the focus of future research, especially in sirtuins and lncRNA. In summary, this study provides valuable information for summarizing research progress and exploring future research directions.

## Data Availability

The original contributions presented in the study are included in the article/Supplementary Material, further inquiries can be directed to the corresponding author.
